# Fatal Delay in a Case of Obstetrics

**Published:** 1892-11

**Authors:** H. F. Jackson

**Affiliations:** Phœnix City, Ala.


PHŒNIX CITY, Ala., Aug. 26, 1892.

### FATAL DELAY IN A CASE OF OBSTETRICS.

I was called to see a lady on the 12th inst. who had been in labor seventy-two hours. She lived five miles from this place. I arrived about eight o'clock at night and found an old midwife and a so-called doctor present. The "doctor" invited me in to take supper, but I declined until I could see what was the patient's condition. This was found to be very weak and exhausted. On examination the lower pelvis proved to be empty, and the os being dilated, I carried my hand into the uterus and found a large hydrocephalic child. The feet were brought down and the body of the child quickly delivered. The head could not pass. I therefore opened it with instruments, the water escaped and delivery was readily accomplished. The time occupied in the entire operation was thirty minutes. The woman died, but I will always think that if she could have been delivered even four hours sooner she would be alive to-day. I hope the people will soon find out the difference between midwives, quacks and doctors.

H. F. JACKSON.

---

### APPENDICITIS.

*General Conclusions*—1. Never adopt the rule of making operative interference the last resort in the treatment of disease peculiar to the right iliac fossa.

2. The only genuine safety for a patient with a diseased vermiform appendix is to have it placed in a bottle of alcohol for the adornment of some surgeon's pathological museum.

3. Early operation in the disease of the vermiform appendix is the only safeguard against recurrent attacks of inflammation, perforation, suppuration and death.

4. Never wait for suppuration in attacks of acute appendicitis. Such practice is just as sensible as it is to wait for stercoraceous vomiting and gangrene of the bowel before operating in strangulated hernia ; for, like in strangulated hernia, procrastination is the thief that has stolen thousands of patients from the physician and surgeon, and given them to the undertaker.—REED, *Lancet-Clinic.*

